# Attitudes and Relationship between Physicians and the Pharmaceutical Industry in a Public General Hospital in Lima, Peru

**DOI:** 10.1371/journal.pone.0100114

**Published:** 2014-06-30

**Authors:** Aldo De Ferrari, Cesar Gentille, Long Davalos, Leandro Huayanay, German Malaga

**Affiliations:** 1 Facultad de Medicina Alberto Hurtado, Universidad Peruana Cayetano Heredia, Lima, Peru; 2 Departamento de Medicina, Hospital Nacional Cayetano Heredia, Lima, Peru; Johns Hopkins University, United States of America

## Abstract

**Background:**

The interaction between physicians and the pharmaceutical industry influences physicians' attitudes and prescribing behavior. Although largely studied in the US, this topic has not been well studied in resource-poor settings, where a close relationship between physicians and industry still exists.

**Objective:**

To describe physician interactions with and attitudes towards the pharmaceutical industry in a public general hospital in Lima, Peru.

**Design:**

Descriptive, cross-sectional study through an anonymous, self-filled questionnaire distributed among faculty and trainee physicians of five different clinical departments working in a Peruvian public general hospital. A transcultural validation of an existing Spanish questionnaire was performed. Exposure to marketing activities, motivations to contact pharmaceutical representatives and attitudes towards industry were studied. Collected data was analyzed by degree of training, clinical department, gender and teaching status. Attitudes were measured on a four-point LIKERT scale.

**Results:**

155 physicians completed the survey, of which 148 were included in the study sample. 94.5% of attending physicians reported ongoing encounters with pharmaceutical representatives. The most common industry-related activities were receiving medical samples (91.2%), promotional material (87.8%) and attending meetings in restaurants (81.8%). Respondents considered medical samples and continuing medical education the most ethically acceptable benefits. We found significant differences between attendings and residents, and teaching and non-teaching attendings. An association between the amount of encounters with pharmaceutical representatives, and attitudes towards industry and acceptance of medical samples was found.

**Conclusions:**

A close physician-industry relationship exists in the population under study. The contact is established mainly through pharmaceutical representatives. Medical samples are the most received and ethically accepted benefit. The attitudes of physicians on the ethical standards of acceptance of medical samples and other benefits are closely related with their exposure to the pharmaceutical industry. Future studies could explore the motivations of physicians working in resource-poor settings to maintain a close relationship with industry.

## Introduction

The interaction and potential conflicts of interest between physicians and the pharmaceutical industry has been noted in recent years [1], [2]. The concern among researchers and the general public is that the primary interest of the practitioner, the patient's well-being, might be influenced by secondary interests like benefits offered by the pharmaceutical industry to physicians [3]. In the last decade, total marketing expenditures by pharmaceutical companies equaled the amount invested in research and development of new drugs [4], which further raises suspicion of potential conflicts of interest. Marketing activities include detailing, provision of gifts to physicians, sponsorship of social activities and support of educational and research undertakings [5].

Wazana et al. showed that the interaction between physicians and the pharmaceutical representatives adversely influences doctor's behavior and knowledge [6]. They report a tendency for non-rational prescribing, more favorable attitudes towards industry representatives, preference for newer, more expensive drugs and an inability to identify incorrect claims about medications. Moreover, this effect on physicians' behavior and knowledge is dose-dependent: frequent contacts and acceptance of gifts (independent of the gift's value [5]) imposes a need to reciprocate that affects medical judgment [7].

Physicians do not seem to be aware of the effect of marketing activities on their own conduct. Korenstein showed that doctors tend to recognize that their colleagues' prescribing, but not their own, is affected by industry's promotion [8]. Likewise, the influence of marketing also affects the attitudes and behaviors of doctors-in-training [6] [9], [10]. On the other hand, one study performed in Spain suggests that physicians might be aware of the effect of marketing on their behavior, but they consider marketing activities ethically acceptable if the patients end up benefiting from them [11].

Although the physician-industry relationship has been vastly described in the literature, this topic has not been well studied in resource-poor settings. It is a common belief that physicians can actually help their economically less-favored patients by giving them medical samples or participating in industry-funded CME activities. However, evidence suggests that non-compliance with treatment and patient's inability to pay for medications is more common among physicians with a close relationship with the industry than among those with less frequent contact [12]. It is therefore important to understand the reasons and beliefs that motivate a close doctor-industry relationship despite growing evidence showing the potential harms that may arise from this relationship.

This work seeks to describe physician's interaction with and attitudes towards industry in a general hospital from a marginal district in Lima, Peru. For this purpose, we performed a transcultural validation of a Spanish questionnaire on this topic [11] and distributed it among faculty and trainee physicians working at the mentioned hospital.

## Methods

### Ethics Statement

The study was reviewed and approved by the ethics committees of the Universidad Peruana Cayetano Heredia and of Hospital Nacional Cayetano Heredia in Lima, Peru.

### Study Design

We performed a descriptive, cross-sectional study. In the first phase, we did a transcultural validation from an already existing questionnaire developed by Galán Herrera et al., following the guidelines for the cross-cultural adaptation process developed by Beaton et al. [13]. We modified and substituted questions and terms from the original questionnaire to adapt them to our context. Five experts (one Internal Medicine Doctor with vast experience in bioethics, a psychologist, the medical director of a pharmaceutical company, an epidemiologist and a research physician) evaluated the survey. Following their suggestions, we prepared a new version of the questionnaire. A pilot study was performed on 20 physicians (ten attendings and ten residents) working at a similar public general hospital from Lima. The final version of the questionnaire included data obtained from the pilot study.

In the second phase of the study, we administered the survey to 155 attendings and residents from the departments of Medicine, Surgery, Pediatrics, Gynecology and Obstetrics, and Anesthesiology from Hospital Nacional Cayetano Heredia, which was selected for being a reference, teaching general hospital. This center serves a marginal, un- or underemployed population of Lima that may qualify to receive a free government-funded insurance that covers a limited bundle of diseases and services. Eligibility criteria included being an attending or resident at Hospital Nacional Cayetano Heredia working in one of the five departments mentioned above during March of 2013. Participants were selected by a non-probability convenience sampling technique.

After assenting to written informed consent, each participant was given a paper-based copy of the questionnaire to be returned in a closed envelope. The survey was anonymous and self-filled, and consisted of an introductory page and 49 questions organized in 5 sections: (1) general information, e.g. gender, age and specialty, among others; (2) physicians' exposure to industry's promotion activities (13 questions), e.g. drug detailing frequency, number of gifts and promotional material received etc.; (3) motivation for seeing industry representatives (1 question); (4) physicians' attitudes towards industry (31 questions), evaluated using a four-point Likert scale; and (5) physicians' behavior when presented with an everyday scenario regarding interaction with industry (4 questions).

### Data Analysis

We calculated frequencies, means and standard deviations (SD) and used chi-square test with Yates' correction to determine statistical differences between categorical variables. We considered a p-value≤0.05 as statistically significant. We conducted statistical analysis in R (www.r-project.org).

### Search strategy

We performed a literature search of Pubmed (National Library of Medicine, Bethesda, MD), Scielo, Cochrane Library and Google (1994–2014) combining the following keywords (*pharmaceutical industry* [text word] OR *physicians* [text word] O*R gifts* [text word] OR *survey* [text word] OR *resource poor setting* [text word], or the names of specific countries) and included studies in English, Spanish and Portuguese. We selected studies that surveyed attendings or residents of any specialty, and that reported at least one of the following: frequency or percentage of physicians (1) having contact with pharmaceutical representatives, (2) receiving medical samples, and/or (3) receiving other gifts or benefits from industry. We excluded studies directed to medical students and those that did not report the outcomes of interest.

## Results

The study sample consisted of 155 attendings and residents working at a Peruvian public general hospital during March of 2013. Seven participants were excluded from the analysis because they did not return the questionnaire or left 5 or more questions unanswered. A total of 148 participants (95.5% response rate) were included in the analysis. The mean ± SD age was 38±10 years, 65% of the participants were men and 61% were attendings (Table 1).

**Table 1:** Characteristics of study participants.

Characteristics	Value (%) n = 148
**Age**	
Total simple	38±10 years*
Attendings	43±10 years*
Residents	31±5 years*
**Position** †	
Attendings	91 (61.5%)
Residents	56 (38.5%)
**Teaching status** ‡	
Teaching Attendings	54 (60%)
Non-teaching Attendings	36 (40%)
**Gender**	
Male	96 (64.9%)
Female	52 (35.1%)
**Specialty**	
Internal Medicine and subspecialties	59 (39.9%)
Attendings	31 (52.5%)
Residents	28 (47.5%)
General Surgery and subspecialties†	32 (21.6%)
Attendings	21 (67.7%)
Residents	10 (32.3%)
Pediatrics	28 (18.9%)
Attendings	14 (50%)
Residents	14 (50%)
Obstetrics and Gynecology	16 (10.8%)
Attendings	13 (81.2%)
Residents	3 (18.8%)
Anesthesiology	13 (8.8%)
Attendings	12 (92.3%)
Residents	1 (7.7%)

*Mean ± standard deviation.

† One physician (General Surgery and subspecialties) did not report her position.

‡ One attending did not report his teaching status.

The study showed that there is an active relationship between physicians and the pharmaceutical industry in the studied population. Ninety five percent of attendings and 85.7% of residents indicated having at least one monthly interaction with pharmaceutical representatives. In these encounters, 91.2% of participating physicians received medical samples and 87.8% promotional material. Furthermore, 81.8% of practitioners accepted invitations to dinners or cocktails at local restaurants with a frequency of at least once every four months. In contrast, receiving material or equipment for professional use (31.8%), personal gifts (27.8%) and travel expenses (e.g. lodging) to congresses (15.5%) were considerably less common. The main reasons for seeing pharmaceutical representatives were “out of respect for another's job” (81.1%) and “to obtain medical samples for my patients” (55.2%).

Regarding the attitudes of physicians towards industry's detailing, 75% considered that the information brought up by pharmaceutical representatives is “not trustworthy” and 80.3% stated that reps “prioritize the promotion of their products over patients' benefit”. In spite of this, 47.6% of all participants stated that the information provided by pharmaceutical representatives helps them “learn about new products” and “stay up to date”. The majority of surveyed doctors (88.5%) think that receiving gifts or lunches from industry has no influence on their prescribing behavior, and a smaller proportion (35.2%) think that those same gifts affect their colleagues' prescriptions. With respect to the influence on physicians' prescribing behavior, five activities were regarded as potentially conditioning: funding of registration costs to conferences (50.3%), funding of lodging during congresses (51.7%) or of continuing medical education (CME) (53.1%), attending promotional travels (53.4%) and participating in or receiving funds for research (54.8%). The most ethically perceived activities were receiving medical samples (81.8%) and continuing medical education (68.9%) (Table 2).

**Table 2:** Percentage of participants who agree or disagree with the following statements.

	Agree	Disagree
**Statements**		
The information provided by pharmaceutical representatives helps me stay up to date or learn about new products	47,6%	52,4%
The information provided by pharmaceutical representatives is trustworthy	25,0%	75,0%
Pharmaceutical representatives prioritize the promotion of their products over patients' benefit	80,3%	19,7%
Receiving gifts or meals affects my prescribing	11,5%	88,5%
Receiving gifts or meals affects my colleagues' prescribing	34,9%	65,1%
**Receiving these materials or participating in these activities affects doctors' prescribing behavior**		
Promotional materials (food, pens, etc.)	34,0%	66,0%
Medical samples	48,3%	51,7%
Gifts not related to medical practice valued at <US$50	31,5%	68,5%
Gifts not related to medical practice valued at >US$50	41,2%	58,8%
Cocktails, lunches or dinners	44,9%	55,1%
Funding of registration costs to conferences	50,3%	49,7%
Continuing medical education	53,1%	46,9%
Participation in industry-funded research (clinical trials, case-control studies, cross sectional studies etc.)	54,8%	45,2%
**Receiving these materials or participating in these activities is ethical**		
Promotional materials (food, pens, etc.)	65,5%	34,5%
Medical samples	81,8%	18,2%
Gifts not related to medical practice valued at <US$50	27,0%	73,0%
Gifts not related to medical practice valued at >US$50	22,3%	77,7%
Cocktails, lunches or dinners	51,7%	48,3%
Funding of registration costs to conferences	60,8%	39,2%
Continuing medical education	68,9%	31,1%
Participation in industry-funded research (clinical trials, case-control studies, cross sectional studies etc.)	64,6%	35,4%

Compared to residents, attendings reported a greater number of monthly encounters with pharmaceutical representatives and receiving a larger amount of medical samples and promotional material (Table 3). Similarly, attendings were more prone to believe that gifts and lunches do not influence their prescribing behavior (42.2% vs. 23.6%; p = 0.036). We found no significant differences in the amount of encounters with pharmaceutical representatives between men and women, neither among attendings (51.6% of men and 40.7% of women reported >10 encounters per month; p = 0.47) nor among residents (9% of men and no women reported >10 encounters per month; p = 0.62).

**Table 3:** Participation in activities and acceptance of gifts from industry stratified by position (attendings vs. residents).

	Attendings	Residents	p-value
**Monthly encounters with pharmaceutical representatives**			
≥5 encounters	67 (73.6%)	11 (19.6%)	<0.001
**Number of occasions in which medical samples are received in a month**			
≥5 occasions	62 (68.1%)	9 (16.1%)	<0.001
**Number of occasions in which promotional material is received in a month**			
≥5 occasions	35 (38.5%)	6 (10.7%)	<0.001
**Registration to conferences financed by the pharmaceutical industry in a year** †			
≥1 registration‡	35 (38.9%)	17 (30.9%)	0.427

† One teaching attending did not answer this question.

Among attendings, those involved with teaching reported a higher number of monthly encounters with industry representatives, receiving more medical samples and more invitations to attend conferences than their non-teaching counterparts (Table 4). Additionally, they tended to believe that medical samples do not affect prescribing patterns (33.3% vs. 58.3%; p = 0.03) and that it is ethically appropriate to receive promotional material (81.5% vs. 58.3%; p = 0.03).

**Table 4:** Participation in activities and acceptance of gifts from industry stratified by teaching status.

	Teaching attendings	Non-teaching attendings	p-value
**Monthly encounters with pharmaceutical representatives**			
≥5 encounters	46 (85.2%)	20 (55.6%)	0.004
**Number of occasions in which medical samples are received in a month**			
≥5 occasions	43 (79.6%)	18 (50%)	<0.001
**Number of occasions in which promotional material is received in a month**			
≥5 occasions	23 (42.6%)	11 (30.6%)	0.351
**Registration to conferences financed by the pharmaceutical industry in a year** †			
≥1 registrations	26 (49.1%)	8 (22.2%)	0.019

† One teaching attending did not answer this question.

From the studied specialties, anesthesiologists reported the least number of encounters with pharmaceutical representatives (Table 5) and more than half of them felt that most promotional activities and gifts affect physicians' prescribing behavior. In contrast to anesthesiologists, pediatricians reported the largest number of monthly encounters with industry representatives and similarly more than half of them did not believe that promotional activities and gifts have an impact on prescribing patterns. Given that the amount of participants in some specialties was small (e.g. anesthesiology), we could not determine if the differences found among specialties were statistically significant.

**Table 5:** Participation of attendings in activities and acceptance of gifts from industry stratified by specialty.

	Internal Medicine and subspecialties	Surgery and subspecialties	Pediatrics	Obstetrics and gynecology	Anesthesiology
**Monthly encounters with pharmaceutical representatives**					
<5 encounters	5 (16.1%)	5 (23.8%)	0 (0%)	3 (23.1%)	11 (91.7%)
≥5 encounters	26 (83.9%)	16 (76.2%)	14 (100%)	10 (76.9%)	1 (8.3%)
**Number of occasions in which medical samples are received per month**					
<5 occasions	7 (22.6%)	7 (33.3%)	0 (0%)	3 (23.1%)	12 (100%)
≥5 occasions	24 (77.4%)	14 (66.7%)	14 (100%)	10 (76.9%)	0 (0%)
**Receiving promotional material affects prescribing behavior**	12 (38.7%)	5 (25%)*	2 (14.3%)	3 (23.1%)	7 (58.3%)
**Receiving medical samples affects prescribing behavior**	14 (45.2%)	9 (42.8%)	2 (14.3%)	5 (38.5%)	9 (75%)
**Receiving gifts not related to medical practice valued at <US$50 affects prescribing behavior**	11 (35.5%)	5 (23.8%)	2 (14.3%)	4 (30.8%)	6 (50%)
**Funding of registration costs to conferences affects prescribing behavior**	17 (54.8%)	10 (47.6%)	3 (21.4%)	5 (38.5%)	8 (66.7%)

*One participant did not answer that question.

In the clinical vignette, more than 82% of participants were willing to accept medical samples to offer them as free treatment to their resource-poor patients. However, only 58% accepted the offer when asked to give talks to other physicians supporting the company's products, and only 9% accepted if forced to prescribe other drugs from the same company. Interestingly, from the 118 physicians who would accept samples for their patients, 88% considered that it was ethical to receive medical samples, while only 57% had the same opinion (p<0.001) among the 30 participants that were not willing to accept the treatment.

Our data suggests that there is a negative relationship between the amount of exposure to industry's promotional activities and reporting that gifts, lunches and other benefits affect prescribing behavior (Table 6).

**Table 6:** Attitudes of physicians (attendings and residents) towards industry stratified by number of encounters held with pharmaceutical representatives.

Statements	<5 encounters	n	≥5 encounters	n	P-value
Receiving medical samples affects prescribing behavior	48 (69.6%)	69	23 (29.4%)	78	<0,001
Receiving invitations to cocktails, lunches or dinners affects prescribing behavior	38 (54.3%)	70	28 (36.4%)	77	0,043
Receiving promotional material affects prescribing behavior	32 (47.1%)	68	17 (22.4%)	76	0,003
Receiving material related to medical practice (otoscopes, vademecums) affects prescribing behavior	37 (53.6%)	69	22 (28.2%)	78	0,003
Receiving learning material (text books) affects prescribing behavior	38 (54.3%)	70	24 (30.8%)	78	0,006
Receiving gifts not related to medical practice valued at <US$50 affects prescribing behavior	28 (41.2%)	68	18 (23.1%)	78	0,03
Funding of registration costs to conferences affects prescribing behavior*	44 (63.8%)	69	30 (38.5%)	78	0,006
Industry-funded CME affects prescribing behavior	46 (66.7%)	69	32 (41%)	78	0,005

*Cut-off point was 10 encounters instead of 5 as in previous examples.

## Discussion

Our results suggest that a close relationship exists between physicians and the pharmaceutical industry in the studied population. The vast majority of surveyed doctors reported having ongoing encounters with pharmaceutical representatives, which explains why medical samples and promotional material were the most common gifts accepted from industry. In contrast, other activities such as receiving funding for medical congresses or lodging and taking part in research undertakings were considerably less common.

Studies from different parts of the world report variable results regarding the interaction with industry (Appendix 1). For example, a German survey among physicians in private practice reports that 92% of the doctors received medical samples [14], while only 41% of faculty in an American study accepted them [15]. Regarding the practices of accepting gifts from industry and having interactions with pharmaceutical representatives, results range from 52% to 83% [16], [17] and from 47% to 98% [18], [19] for each of these activities respectively.

There were no significant differences between teaching and non-teaching attendings or between attendings and residents regarding their motivations for seeing pharmaceutical representatives. Most of these results coincide with those reported by Galán Herrera et al. among Spanish primary-care physicians [11]; in both studies, “respect for another's job” was the main reason for seeing pharmaceutical representatives. However, another important reason reported in that study, “to learn about new drugs”, was not important in our research, probably due to the fact that 75% of respondents did not consider the information provided by the pharmaceutical industry to be trustworthy.

The great majority of participants reported that accepting gifts or lunches does not affect their prescribing habits. Similar results have already been described by Valenzuela-Rodríguez in Peru [20]. In his study, Internal Medicine physicians did not consider that marketing activities or the literature provided by pharmaceutical representatives influenced their prescribing behavior. However, in our study, more than one third of all surveyed physicians believed that their colleagues were more likely to be influenced by gifts and meals from industry than they were. Such discrepancies have been described in similar studies [8], which suggests that physicians are not aware of the impact that industry's marketing activities have on their behavior. Interestingly, our results show that attendings were more prone to this kind of thinking than residents were. This lack of awareness might explain why the majority of physicians report ongoing relationships with the industry, despite questioning the quality and trustworthiness of the information provided by pharmaceutical representatives.

In spite of the favorable attitudes held towards several interactions with industry, nearly half of all participants recognized that some practices, e.g. receiving medical samples or taking part in research financed by industry, influence their prescribing patterns. This has been reported in several studies that show an increase in prescriptions of promoted drugs immediately after providing medical samples to physicians or inviting them to congresses and/or promotional trips [6], [21].

The majority of participants in our study pointed out that it is ethical to receive medical samples or to accept invitations to congresses or CME programs, among other activities. This shows a contradiction among the study participants because most of them considered these activities as influencing physicians' prescribing behavior. Galán Herrera et al. proposed an explanation for this phenomenon. They argue that physicians are aware that these practices influence their prescribing patterns, but they consider them ethical if the patient is benefited (e.g. by receiving medical samples) [11]. Our results suggest the same line of thought: physicians working in resource-poor settings view medical samples as a tangible benefit for their patients and consider that receiving them is ethically correct, while accepting personal gifts (e.g. unrelated to the medical practice) is not.

Attendings reported a significantly greater interaction with industry than residents did. In comparison with other studies [6], [17], our data shows more encounters with pharmaceutical representatives and more acceptance of medical samples and promotional material among attendings, while the opposite was true among residents. We hypothesize that these differences are secondary to a smaller number of contacts between pharmaceutical representatives and residents (and hence less opportunities to receive gifts) and less time to attend courses or congresses due to the fact that they usually have a greater load of work than attendings. In contrast to previous reports showing significant differences between men and women [11], [17], [22] (with men generally having more favorable attitudes towards industry), our results indicate that there are no major differences between genders in the study population, both among attendings and residents.

Teaching attendings reported a greater closeness to the industry than non-teaching physicians. Literature about this topic shows opposing results. Galán Herrera et al. [11] found that teaching physicians were more critical with industry than non-teaching ones. In contrast, a study performed among faculty members of the Society for Academic Emergency Medicine in the US reported that a higher academic degree correlated with a closer relationship with industry (e.g. receiving more drug samples or tickets to sporting or cultural events) [15]. Our findings are similar to those of the latter study. A possible explanation for our observations is that those doctors involved in teaching activities are (1) more likely to be involved in research projects (sometimes in collaboration with drug companies), and (2) opinion leaders with greater influence on their colleagues, residents and medical students. These characteristics might render them more attractive to the pharmaceutical industry and hence more prone to have a closer relationship with it. Additionally, many medical schools in the US have banned industry representatives from their hospitals, which further raises awareness among teaching physicians about the potential conflicts of interest that might arise from having contact with them. In Peru, teaching physicians work at public hospitals, and universities have no power to limit or ban the presence of industry representatives in those centers. More research is needed to understand the reasons for the differences seen between teaching and non-teaching physicians.

As previously reported by Campbell et al. [17], anesthesiologists in our study reported a smaller exposure to the industry and, in contrast to other specialties, considered that all activities or gifts provided by pharmaceutical companies influence prescribing patterns. This phenomenon could be explained by the fact that many of them have less clinic hours than physicians from other specialties and therefore less frequency of exposure. Moreover, they can evaluate a drug's effectiveness during the anesthetic procedure which makes them more prone to prescribe a drug based on its actual performance rather than on marketing claims.

Pediatricians lay on the other side of the spectrum, having more contacts with pharmaceutical representatives and showing less critical attitudes towards industry. These results differ from those reported by Korenstein et al. In their study, they found more critical attitudes towards industry among pediatricians and internal medicine doctors, and less critical opinions among surgeons and gynecologists [8]. One possible explanation for this difference is that, in our study, pediatricians reported more exposure to industry than surgeons, which usually is associated with more positive attitudes [6].

Our results found two positive associations: one between the attitudes of participants towards industry and the number of encounters with pharmaceutical representatives; and the other between the attitude of respondents towards industry and the quantity of medical samples that they received. These correlations can be interpreted in two contrasting ways. The first interpretation suggests that a positive view of the pharmaceutical companies' promotional activities leads to a closer contact between physicians and the industry. Practitioners whose underlying attitudes towards the industry are positive have a closer relationship with representatives of the industry. Alternatively, some doctors might consider that receiving medical samples favors their resource-poor patients [23] and hence report a positive view of meeting with industry's representatives, despite having a general negative attitude towards industry. The second interpretation views the aforementioned associations in the opposite way: closer and frequent encounters with pharmaceutical representatives lead to physicians having a more positive image of the industry. As described by Wazana [6], doctors and medical students who have frequent contacts with pharmaceutical representatives report more positive attitudes towards industry. Furthermore, this influence also affects prescribing patterns. Chew et al. [24] and Warrier et al. [25] showed that the availability of medical samples affects doctors' prescription patterns without them being aware of this change.

The importance of understanding the effect of attitudes on number of contacts is that permissive attitudes, even if well meant, end up influencing medical practice and prescribing behavior. For example, we found that those who considered that receiving medical samples was ethical were more prone to accept medical samples for their patients in the presented clinical vignette. This influence on medical practice might not always have the desired effect on patients. For instance, many physicians believe that giving medical samples favors their patients, mainly those economically less favored. However, many studies show that patients that receive medical samples usually end up paying higher amounts of money for their medications in the long run [26]. This phenomenon, which is not exclusive to developing countries, can have a considerable impact on a nation's more vulnerable groups, particularly among uninsured patients with chronic diseases. The reason for this is that, once a treatment proved to be effective, patients (and doctors) are reticent to changing medication, leading to more out-of-pocket expenses or treatment discontinuation. The important effect of attitudes on behavior (or vice versa) is key to better understanding industry's marketing strategies: small gifts do not produce a big, immediate increase in prescription patterns; instead, they help to build a long lasting relationship between physicians and the pharmaceutical industry [27]. Additionally, attendings and residents may fail to realize that receiving promotional gifts, medical samples or industry-funded trips increases drug's prices. Taking into account that pharmaceutical companies invest important amounts of money in marketing activities, it would be in the patient's best interest to stop receiving gifts and medical samples, so that the price of drugs would be more affordable.

Our study has some potential shortcomings. First, the study participants might have underreported their interactions with the pharmaceutical industry in order to present themselves in a better light (social desirability bias). We tried to reduce this bias by using anonymous questionnaires returned in closed envelopes. Second, this study was conducted in one teaching hospital from Lima, which limits generalizability to other settings. Third, we used a non-probability convenience sampling technique, which may lead to sampling bias.

In conclusion, our results show that there exists a close and active relationship between physicians and the pharmaceutical industry in the context of a public general hospital in Lima, Peru. The main form of contact is through pharmaceutical representatives with a high number of physicians reporting ongoing monthly encounters. Medical samples were the most received and ethically accepted benefit while personal gifts were considered least ethical. Our findings suggest that, in the setting of a marginal general hospital in Peru, physicians seek palpable/material benefits for their patients and have poor awareness of the conflicts of interest and harms that may arise from this relationship, even in an academic environment. Future studies could explore the motivations of physicians working in resource-poor settings to maintain a close relationship with industry, and the role of education and legislation in these settings.

## Supporting Information

Appendix S1 **Literature from other countries describing the physician-industry relationship from 1994 to 2014.** We included studies that reported at least one of the following: (1) having contact with pharmaceutical representatives, (2) receiving medical samples, and (3) receiving other gifts or benefits from industry.
(DOCX)
