# Supplementary material for: Attitudes and Relationship between Physicians and the Pharmaceutical Industry in a Public General Hospital in Lima, Peru
Source: PLoS One. 2014 Jun 30;9(6):e100114. doi: 10.1371/journal.pone.0100114 (PMC4076259; doi:10.1371/journal.pone.0100114)
Supplement: Appendix S1 — Literature from other countries describing the physician-industry relationship from 1994 to 2014. We included studies that reported at least one of the following: (1) having contact with pharmaceutical representatives, (2) receiving medical samples, and (3) receiving other gifts or benefits from industry. (DOCX) [file pone.0100114.s001.docx]

**Appendix 1. Literature from other countries describing the physician-industry relationship from 1994 to 2014.**

| **Author** | **Year** | **Country** | **Sample** | **Findings** |
| --- | --- | --- | --- | --- |
| Al-Areefi et al. | 2013 | Yemen | 32 physicians from private and public hospitals. | Frequency of interactions with PRs: 5 per week (median). |
| Alssageer and Kowalski | 2012 | Libya | 608 primary and secondary care providers in public, private and university hospitals. | Physicians that reported interactions with PRs: 47% at least once in the last month and 94% at least once in the last year.  Physicians that accepted medical samples: 69% at least once in the last year.  Physicians that accepted gifts: 73% at least once in the last year. |
| Lobo et al. | 2012 | Spain | 659 physicians of public and private practice. | Physicians that reported interactions with PRs: 90% at least once per month. |
| Fiaschetti et al. | 2011 | Brasil | 127 physicians. | Physicians that reported interactions with PRs: 80% at least once per week. |
| Lieb and Brandtonies | 2010 | Germany | 208 physicians from private practice working in primary care or in the Departments of Neurology, Psychiatry and Cardiology. | Physicians that reported interactions with PRs: 77% at least once per week.  Physicians that accepted medical samples: 92%*.  Physicians that accepted gifts: 96%*. |
| Sharma et al. | 2010 | India | 80 residents and interns (medical specialty not specified). | Physicians that accepted gifts: ≥83% in the last 2 years. |
| Schramm et al. | 2010 | Denmark | 47 general practitioners that regularly interact with PRs. | Frequency of interactions with PRs: 22.3 times (mean) during a 6-month period. |
| Saito et al. | 2010 | Japan | National survey of 2621 practicing physicians. | Physicians that reported interactions with PRs: 98% at least once per month.  Physicians that accepted medical samples: 85% at least once per month.  Physicians that accepted stationary: 96% at least once per month. |
| Garces et al. | 2009 | Spain | 63 primary care physicians. | Physicians that reported interactions with PRs: 77.8% daily. |
| Wang and Adelman | 2009 | USA | 122 ophtalmology residents. | Physicians that reported interactions with PRs: 87% at least once every 1-2 months. |
| Birkhahn et. al | 2008 | USA | 430 faculty members of the Society for Academic Emergency Medicine. | Physicians that reported interactions with PRs: 82% at least once in the last year.  Physicians that accepted medical samples: 41% at least once in the last year.  Physicians that accepted gifts: 73% at least once in the last year. |
| Campbell et al. | 2007 | USA | 1662 physicians from the Departments of Cardiology, Internal Medicine, Family Medicine, Surgery and Pediatrics. | Physicians that accepted medical samples: 78% at least once in the last year.  Physicians that accepted gifts: 83% at least once in the last year. |
| Fagundes et al. | 2007 | Brasil | 25 general physicians and 25 surgeons. | Physicians that reported interactions with PRs: 12% daily, 52% at least once per week and 74% at least once per month.  Physicians that accepted gifts: 86%*. |
| McNeill et al. | 2006 | Australia | 823 medical specialists. | Physicians that accepted items for the office: 94% at least once in the last year.  Physicians that accepted personal gifts: 52% at least once in the last year. |
| Castresana et al. | 2005 | Argentina | 78 physicians (residents and attendings) from the departments of Internal Medicine, Cardiology and Dermatology working in ambulatory settings of public and private hospitals. | Physicians that accepted medical samples: 86%*.  Physicians that accepted stationary: 39%*. |
| Gravalos et al. | 2001 | Spain | 104 primary care physicians regularly interact with PRs. | Frequency of interactions with PRs: 2.8 times per day (mean). |
| Güldal and Semin | 2000 | USA | 446 physicians. | Physicians that reported interactions with PRs: 53.9% at least once per day. |
| Ferguson et al. | 1999 | USA | 346 internists working in a medical school hospital and two affiliated community hospitals. | Physicians that reported interactions with PRs: 83% at least once in the last year.  Physicians that accepted medical samples: 72% at least once during the last year. |
| Strang et al. | 1996 | Canada | 262 physicians in all settings. | Frequency of interactions with PRs: 4.2 times per week (mean). |
| Thomson et al. | 1994 | New Zeland | 67 general practitioners. | Physicians that reported interactions with PRs: 87% with a frequency of 3-4 times per month. |

*Time frame not specified.

Abbreviations: PR, pharmaceutical representative

**REFERENCES**

1. Al-Areefi M, Hassali M, Izham M, Ibrahim M (2013) Physician’s perception of medical representatives visits in Yemen: a qualitative study. BMC Health Services Research. August, 13:331.
2. Alssageer M, Kowalski R (2012) A survey of pharmaceutical company representative interactions with doctors in Libya. Lybian J Med, 7: 18556.

# Lobo E, Rabanaque M, Carrera P, Abad J, Moliner J (2012) Relationship between physician and industry in Aragon. Gac Sanit. Jul-Aug; 26(4):336-42.

# Fiaschetti M, Mastroianni P, Galduroz J, Loffredo L, Chin C. Opiniões e atitudes do médicos frente às ações promocionais da indústria farmacéutica (2011) Revista Baiana de Saúde Pública, v. 35 (4), p. 932-947.

# Lieb K, Brandtonies S (2010) A survey of german physicians in private practice about contacts with pharmaceutical sales representatives. Dtsch Arztebl Int; 107 (22): 392-8.

1. Sharma V, Sourabh A, Harkirat S, Shashank G, Alka S, et al. (2010) Attitudes and practices of medical graduates in Delhi towards gifts from the pharmaceutical industry. Indian Journal of Medical Ethics Vol VII No 4 October-December.
2. Schramm J, Andersen M, Vach K, Kragstrup J, Kampmann J. Promotional methods used by representatives of drug companies: A prospective survey in general practice (2007) Scandinavian Journal of Primary Health Care 25: 93-97.
3. Saito S, Mukohara K, Bito S (2010) Japanese practicing physician’s relationships with pharmaceutical representatives: a national survey. PLoS One 5 (8):e12193.  doi: 10.1371/journal.pone.0012193.
4. Garces G, Colán C, Sanchez A, Gomez G, Canchig F, et al. (2010) Opinión sobre la Visita Médica de los Médicos de Atención Primaria de Toledo. Revista Clínica de Medicina de Familia 3 (1): 5-9
5. Wang Y, Adelman RA (2009) A study of interactions between pharmaceutical representatives and ophtalmology trainees. Am J Ophtalmol. 148 (4): 619-622. doi: 10.1016/j.ajo.2009.05.011.
6. Birkhahn RH, Blomkalns AL, Klausner HA, Nowak RM, Raja AS, et al. (2008) Academic emergency medicine faculty and industry relationships. Acad Emerg Med. 15 (9), 819-824
7. Campbel EG, Gruen RL, Mountford J (2007) A national survey of physician-industry relationships. N Engl J Med. 17, 1742-1750.
8. Fagundes MJ, Gomes-Alves M, Diniz M, Ribeiro J, Garrafa V (2007) [Bioethical analysis of drugs advertisement and publicity]. Cien Saude Colet. 12, 221-229.
9. McNeill PM, Kerridge IH, Henry DA, Stokes B, Hill SR, et al. (2006) Giving and receiving of gifts between pharmaceutical companies and medical specialists in Australia. Intern Med J. 36 (9): 571-578.
10. Castresana L, Mejia R, Aznar M (2005) [The attitude of physicians regarding the promotion strategies of the pharmaceutical industry]. Medicina (B Aires) 65 (3), 247-251.
11. Gravalos G, Palmeiro G, Nuñez E, Casado I (2001) Opinión de los médicos de atención primaria de ourense sobre algunos aspectos de su prescripción farmacéutica. Rev Esp Salud Pública 75, 361-374
12. Güldal D, Semin S (2000) The influences of drug companies' advertising programs on physicians. Int J Health Serv. 30 (3), 585-595.
13. Ferguson R, Rhim E, Belizaire W, Egede L, Carter K, et al. (1999) Encounters with pharmaceutical sales representatives among practicing internists. Am J Med. 107 (2),149-152.
14. Strang D, Gagnon M, Molloy W, Bedard M, Darzins P, et al. (1996) National survey on the attitudes of Canadian physicians towards drug-detailing by pharmaceutical representatives. Ann R Coll Physicians Surg Can. 29 (8), 474-478.
15. Thomson A, Craig B, Barham PM (1994) Attitudes of general practitioners in New Zealand to pharmaceutical representatives. Br J Gen Pract. 44(382), 220-223.
